# Polyamine metabolic enzyme SAT1 remodels the neuronal transcriptome and rescues α-synuclein toxicity in *Drosophila*

**DOI:** 10.1007/s00726-026-03544-y

**Published:** 2026-08-30

**Authors:** Zoya R. Bangash, Hiroyoshi Matsui, Bedri Ranxhi, Sokol V. Todi, Peter A. LeWitt, Wei-Ling Tsou

**Affiliations:** 1 Department of Pharmacology, Wayne State University School of Medicine, 540 E Canfield, Scott Hall Rm 3108, Detroit, MI 48201 USA; 2 Department of Neurology, Wayne State University School of Medicine, Detroit, MI USA

**Keywords:** Autophagy, Mitochondrial quality control, Neurodegeneration, Parkinson’s disease, Polyamine interconversion, RNA sequencing

## Abstract

Polyamine homeostasis is tightly regulated by interconversion and catabolic pathways and has been increasingly implicated in neurodegenerative disorders, including Parkinson’s disease (PD), where accumulation of α-synuclein (α-Syn) perturbs neuronal homeostasis. Spermidine/spermine N¹-acetyltransferase 1 (SAT1) occupies a central position in polyamine interconversion, and alterations in SAT1 activity have been linked to α-Syn toxicity and PD-related neuropathology. To investigate how SAT1 activity influences α-Syn-associated neurodegeneration, we employed a *Drosophila* model of neuronal α-Syn expression. SAT1 overexpression reduced α-Syn protein levels, altered its subcellular distribution within the brain, and mitigated α-Syn-induced lifespan shortening. Transcriptomic analyses showed that SAT1 modulates stress-associated gene expression in the α-Syn background, including attenuation of chaperone and ubiquitin-related responses and coordinated changes in pathways linked to mitochondrial function and amino acid metabolism. SAT1 co-expression partially restored α-Syn-associated alterations in genes involved in mitochondrial quality control, including USP30, Uch-L5R, RNF185, and the mitochondrial ornithine carrier SLC25A15. At the protein level, SAT1 increased mitochondrial-associated signal, enhanced LC3 association with mitochondrial compartments, restored LC3-II/LC3-I ratios in mitochondrial fractions, and reduced mitochondrial accumulation of α-Syn. Functional analyses further showed that SAT1 increased steady-state ATP levels and attenuated the ATP depletion induced by α-Syn expression. These findings indicate that SAT1 activity is associated with reduced α-Syn toxicity and preservation of mitochondrial homeostasis during α-Syn-associated stress.

**Supplementary Information:**

The online version contains supplementary material available at https://doi.org/10.1007/s00726-026-03544-y.

## Introduction

Polyamines (putrescine, spermidine, and spermine) are essential polycations that maintain cellular homeostasis by regulating transcription, translation, bioenergetics, and stress responses (Agostinelli et al. 2010; Vrijsen et al. 2023). In post-mitotic neurons, these metabolites interact with nucleic acids, membranes, and proteins to influence chromatin structure, protein synthesis, membrane properties, and mitochondrial function (Polis et al. 2021; Schibalski et al. 2024). Because neurons depend on long-term proteostasis and organelle integrity, precise control of intracellular polyamine levels is particularly important (Arthur et al. 2024; Dhara et al. 2020; Rhee et al. 2007). Consistent with this vulnerability, age-associated shifts in polyamine balance have been linked to neurodegenerative disorders (Arthur et al. 2024; Malpica-Nieves et al. 2020), including Parkinson’s disease (PD) (Vrijsen et al. 2023). Metabolomic studies report altered polyamine species and acetylated derivatives in PD patient biofluids and tissues, often correlating with disease progression (LeWitt et al. 2023, 2025; Saiki et al. 2019).

**Fig. 1:**
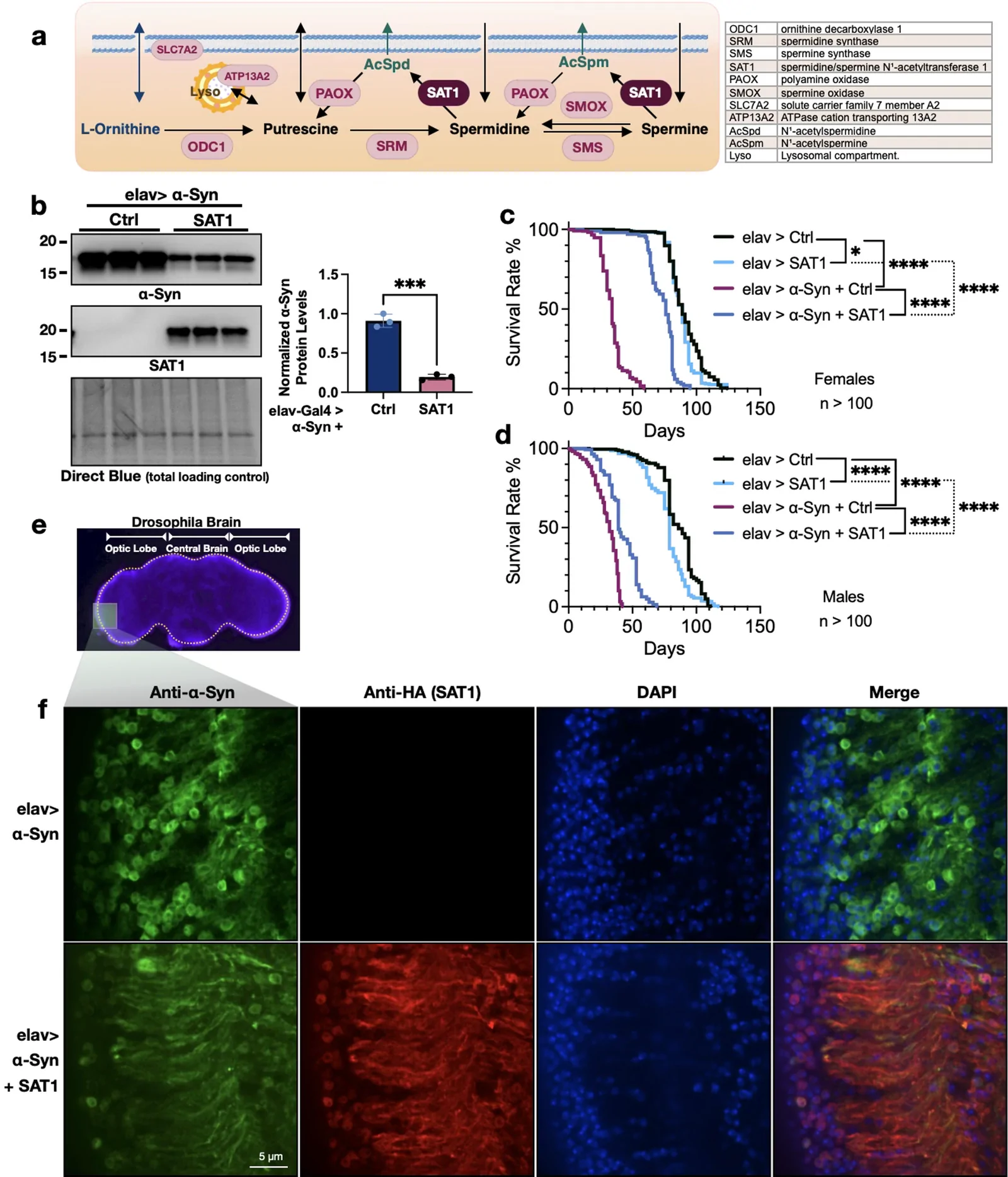
Neuronal expression of SAT1 reduces α-Syn accumulation and restores lifespan in *Drosophila*. **a** Schematic of polyamine metabolism and transport. **b** Western blot analysis of α-Syn protein levels in fly heads expressing α-Syn pan-neuronally in the presence or absence of SAT1. Each biological replicate consisted of 14 heads (7 males and 7 females). Quantified α-Syn levels (right) were normalized to total protein (direct blue). Statistical significance was determined by unpaired two-tailed Student’s t-test. *** *p* < 0.001. **c**, **d** Fly longevity of female (**c**) and male (**d**) flies pan-neuronally expressing Ctrl, SAT1, α-Syn, or SAT1 with α-Syn. Statistical significance was determined by log-rank tests: * *p* < 0.05, **** *p* < 0.0001. **e** Diagram of fly brain regions examined for immunostaining. **f** Representative confocal images of α-Syn and SAT1 immunostaining in the optic lobe of female flies. Scale bar = 5 μm

Polyamine homeostasis is maintained through coordinated biosynthetic, catabolic, and transport pathways (Sagar et al. 2021), with spermidine/spermine N¹-acetyltransferase 1 (SAT1) serving as a key regulator of polyamine catabolic flux (Fig. 1a) (Pegg 2008). SAT1 acetylates spermidine and spermine, promoting their export for further oxidative catabolism and thereby regulating the size and composition of the intracellular polyamine pool (Casero and Pegg 1993). This step is particularly important because, while polyamines can be imported into neurons through specific transport systems, direct efflux is largely restricted to putrescine (Masuko et al. 2003; Uemura et al. 2010). Higher-order polyamines such as spermidine and spermine are inefficiently exported unless first acetylated (Casero and Pegg 2009), placing SAT1 in a unique position to regulate polyamine clearance under stress conditions (Casero et al. 2018). SAT1 expression is strongly inducible by oxidative challenge, inflammation, and toxic insults (Kang et al. 2019; Sui et al. 2021), linking polyamine turnover to cellular stress adaptation. Despite this, SAT1 has been studied primarily in proliferative tissues (Holbert et al. 2022; Nakamura et al. 2021), and its roles in post-mitotic neurons remain incompletely defined.

PD is characterized by the accumulation and aggregation of α-synuclein (α-Syn) (Calabresi et al. 2023), leading to impairments in protein quality control, mitochondrial function, and neuronal survival (Mingo et al. 2025; Thorne and Tumbarello 2022). Increasing evidence links polyamine metabolism to PD-related pathology (Si et al. 2021; Vrijsen et al. 2023). Neuroimaging and postmortem analyses of human PD patients have shown that disease progression affects specific brainstem nuclei early (Lewandowski et al. 2010), with reduced expression of spermidine/spermine N¹-acetyltransferase 1 (SAT1) in PD-vulnerable regions such as the dorsal motor nucleus of the vagus compared with relatively resistant regions, including the olivary nucleus, implicating altered polyamine regulation in region-specific vulnerability (Lewandowski et al. 2010). Consistent with these human observations, transgenic mouse models expressing human α-Syn demonstrate that pharmacologic inhibition of SAT1 with berenil (diminazene aceturate) elevates intracellular spermidine and spermine levels and exacerbates α-Syn aggregation and neuropathology (Lewandowski et al. 2010). In contrast, activation of SAT1 with the polyamine analog DENSPM reduces α-Syn accumulation and ameliorates neurodegenerative phenotypes, implicating SAT1 activity and polyamine flux in modulation of α-Syn pathology (Lewandowski et al. 2010).

At the molecular level, spermidine and spermine can bind α-Syn and accelerate fibril formation in vitro (Antony et al. 2003; Grabenauer et al. 2008; Krasnoslobodtsev et al. 2012), indicating that elevated polyamine levels can promote aggregation under certain conditions. At the same time, spermidine is a well-established inducer of autophagy (Hofer et al. 2024; Watchon et al. 2024), and increased spermidine availability enhances autophagic clearance and reduces α-Syn toxicity in multiple model systems (Buttner et al. 2014; Eisenberg et al. 2009). SAT1, by regulating polyamine interconversion and turnover, is positioned to influence this balance, shaping whether polyamines preferentially contribute to aggregation or engage in protective quality-control pathways. These observations underscore a context-dependent role for polyamines, in which their impact on α-Syn pathology depends on how polyamine flux is regulated within neurons.

In our previous work (Ranxhi et al. 2025b), we established neuronal α-Syn expression in *Drosophila* as a robust in vivo model of PD-relevant toxicity and showed that SAT1 is a potent modifier of disease outcomes: neuronal SAT1 knockdown exacerbated α-Syn pathology, whereas SAT1 overexpression reduced α-Syn levels and conferred strong neuroprotection. The present study extends these findings by defining the molecular mechanisms underlying SAT1-mediated protection. Through transcriptomic and biochemical analyses, we identify SAT1-dependent regulation of pathways involved in protein folding, ubiquitination, mitochondrial quality control, and redox homeostasis under α-Syn stress. Together, these results position SAT1 and polyamine metabolism as central regulators of neuronal stress responses relevant to PD.

## Materials and methods

### Fly lines and genetic crosses

*Drosophila melanogaster* stocks were maintained on standard cornmeal medium at 25 °C and 40% humidity under a 12 h light/12 hr dark cycle. The food consisted of 2% agar, 10% sucrose, 10% yeast, and standard preservatives. Pan-neuronal expression was achieved using the elav-Gal4 driver (BDSC 458), crossed to UAS-SAT1 (Ranxhi et al. 2025b) and UAS-α-synuclein (wild-type human; formerly BDSC 51374). The UAS-α-Syn line (*P{w[+ mC] = UAS-SNCA.J}*) carries an insect codon-optimized human SNCA transgene under UAS control on chromosome 2 and was originally generated by Dr. George Jackson and colleagues. This transgenic line has been widely used in studies of α-Syn-mediated neurodegeneration in *Drosophila* and has been reported in numerous independent publications investigating genetic and molecular modifiers of α-Syn toxicity (Chouhan et al. 2016; Ren et al. 2022; Roy and Jackson 2014; Yu et al. 2023). Although this stock is no longer distributed by the Bloomington *Drosophila* Stock Center, it was obtained from BDSC in 2021 and has since been maintained in our laboratory. Generation and validation of the UAS-SAT1 (CG4210) overexpression line have been described previously (Ranxhi et al. 2025b). Two control lines were used for different experimental purposes. For RNA-seq experiments, the y, w; +; attP2 landing-site line (gift from Jamie Roebuck, Duke University) was used because the UAS-SAT1 transgene was generated at the attP2 genomic insertion site, providing the closest genetic-background match for transcriptomic comparisons involving SAT1 expression. For all other experiments, including lifespan, western blotting, and immunofluorescence, UAS-Luciferase (BDSC 35788) was used as a neutral UAS transgene control under the same GAL4 driver to account for effects associated with expression of an additional UAS transgene.

### Western blotting

Fly heads (7 males + 7 females) were homogenized in hot lysis buffer (50 mM Tris, pH 6.8, 2% SDS, 10% glycerol, 100 mM DTT), sonicated, boiled for 10 min, and centrifuged at maximum speed for 10 min. Protein lysates from at least three biological replicates were resolved by SDS-PAGE using 4–20% Mini-PROTEAN TGX gels (Bio-Rad) and transferred to 0.2 μm PVDF membranes. Membranes were blocked in 5% milk in TBST and incubated overnight at 4 °C with primary antibodies, followed by HRP-conjugated mouse or rabbit secondary antibodies (1:5000, Jackson ImmunoResearch) for 1 h at room temperature. Signals were detected using EcoBright Pico or Femto HRP substrates (Innovative Solutions) and imaged on a ChemiDoc system (Bio-Rad). Membranes were stained with 0.1% Direct Blue 71 for total protein, and α-Syn signal intensity was quantified relative to total lane protein using ImageLab software (Bio-Rad). For mitochondrial fractions, Porin was used as the mitochondrial loading control. Antibodies used were mouse anti-α-Syn (1:1000, ThermoFisher MA1-90346), anti-pS129-α-Syn (1:1000, Cell Signaling Technology 23706), anti-HA (1:1000, Cell Signaling Technology 3724), anti-LC3 (1:1000, Abcam ab109364), anti-K63-linked ubiquitin (1:500, ThermoFisher 14-6077-82), and anti-Porin (1:1000, MilliporeSigma 185–197).

### Adult brain dissection and immunofluorescence

Adult female flies were briefly anesthetized with CO₂, and brains were dissected in ice-cold PBS (pH 7.2). Following removal of the head capsule, brains were fixed in 4% paraformaldehyde for 1 h at room temperature with gentle rotation, washed with PBST, and blocked for 1 h in 2% BSA in PBST. Brains were then incubated overnight at 4 °C with primary antibodies. Primary antibodies used were mouse anti-α-Syn (1:1000, Santa Cruz Biotechnology, sc-12767), rabbit anti-HA (1:1000, Cell Signaling Technology, #3724), mouse anti-ATP5A (1:4000, Abcam, ab14748), and rabbit anti-LC3 (1:1000, Abcam, ab109364). After washing with PBST, tissues were incubated for 2 h at room temperature with Alexa Fluor-conjugated secondary antibodies (goat anti-mouse Alexa Fluor 488 and goat anti-rabbit Alexa Fluor 568, both at 1:2000; Thermo Fisher Scientific). Samples were washed, mounted in ProLong Glass Antifade Mountant (Thermo Fisher Scientific, P36982), and imaged using a Leica SP8 confocal microscope with 63X or 100X objectives.

### Longevity assay

Approximately 20 adult flies, matched by age and separated by sex within 48 h of eclosion as adults from their pupal cases, were collected per vial and maintained on standard cornmeal fly medium at 25 °C. Flies were transferred to fresh food vials every 2 to 3 days, and mortality was monitored daily until all flies had died. Total fly numbers are indicated in each figure. Survival data were analyzed using the log-rank test in GraphPad Prism (San Diego, CA, USA).

### RNA extraction, sequencing, and functional enrichment

Total RNA was extracted from 70 adult fly heads (35 males + 35 females; *n* = 5 biological replicates per genotype) using TRIzol reagent (Thermo Fisher Scientific). mRNA-seq libraries were prepared using the Illumina TruSeq Stranded mRNA Library Prep Kit, incorporating poly(A) selection and strand-specific reverse transcription (AZENTA). Libraries were sequenced on an Illumina NovaSeq platform, generating approximately 20 million 150-bp paired-end reads per sample. RNA-seq data analysis was performed in R (v4.3.1). Raw sequencing reads were processed using fastp (v0.23.1) for adapter trimming and quality control, aligned to the *Drosophila melanogaster* reference genome (BDGP6, FlyBase release FB2023_05) using STAR (v2.5.2b), and quantified at the gene level with featureCounts (Subread v1.5.2). Gene-level count files retaining FlyBase gene identifiers (FBgn) were merged into a single count matrix, and missing values introduced during merging were replaced with zeros. Genes with low expression (total counts ≤ 10 across all samples) were excluded. Raw integer counts were used for differential expression analysis, whereas log₂-transformed counts (log₂[count + 1]) were used for visualization and exploratory analyses. Human gene symbols were appended by merging FBgn identifiers with a curated FlyBase-to-human ortholog mapping table. Differential expression analysis was conducted using DESeq2. Sample group identities were inferred from sample names, and three biologically defined pairwise comparisons were performed: SAT1 vs. Ctrl, α-Syn vs. Ctrl, and α-Syn + SAT1 vs. α-Syn. For each comparison, samples were subset to the relevant groups and the reference condition was explicitly set. Differential expression was assessed using DESeq2’s negative binomial generalized linear model with Wald tests and internal size-factor normalization. Genes with Benjamini-Hochberg adjusted p-values < 0.05 and absolute log₂ fold change > 1 were considered significantly differentially expressed. Global transcriptomic relationships were examined using principal component analysis (PCA), t-distributed stochastic neighbor embedding (t-SNE), and Uniform Manifold Approximation and Projection (UMAP) based on DESeq2 variance-stabilized expression values. Heatmaps were generated using pheatmap with gene-wise scaling and hierarchical clustering. Functional enrichment analyses of up- and downregulated gene sets were performed using the PANGEA platform for Gene Ontology and KEGG pathways. To identify genes whose α-Syn-associated expression changes were reversed by SAT1 co-expression, results from the α-Syn vs. Ctrl and α-Syn + SAT1 vs. α-Syn comparisons were integrated. Reversal genes were defined as those showing opposite directions of regulation between the two comparisons.

### Mitochondria isolation

Mitochondria were isolated from heads of 50 adult (25 males + 25 females) *Drosophila* using the Mitochondria Isolation Kit for Tissue (Thermo Fisher Scientific, #89801), following the manufacturer’s protocol. Briefly, heads were homogenized on ice in isolation buffer and subjected to sequential centrifugation to fractionate cellular components. Samples were first centrifuged at 700 × g to remove insoluble debris (pellet), and the resulting supernatant was centrifuged at 12,000 × g at 4 °C to separate the mitochondrial and cytosolic fractions. Mitochondrial, cytosolic, and pellet fractions were analyzed by Western blot.

### ATP content assay

ATP content was measured using the ATP Determination Kit (Thermo Fisher Scientific, A22066) according to the manufacturer’s instructions with minor modifications for fly heads. Adult flies were separated by sex, and heads were collected and homogenized in ATP extraction buffer containing 100 mM Tris-HCl (pH 7.75) and 4 mM EDTA. For each biological replicate, 10 fly heads were homogenized in 500 µL extraction buffer. Samples were heated at 95 °C, cooled on ice, and clarified by centrifugation. Supernatants were collected and used immediately for ATP measurements. ATP standards were prepared according to the manufacturer’s instructions. For each assay, 10 µL of sample or standard was mixed with 90 µL of reaction mixture containing luciferase and D-luciferin. Luminescence was measured using a TECAN Infinite 200 PRO M Plex microplate reader. ATP concentrations were calculated from the standard curve and normalized to the number of fly heads. ATP content is expressed as pmol ATP per head.

### Statistics

Data are presented as mean ± SEM unless otherwise indicated. Statistical analyses were performed using Prism 9 (GraphPad Software). Survival curves were analyzed using the Kaplan-Meier method and compared by log-rank (Mantel-Cox) tests. For comparisons involving more than two groups, ordinary one-way ANOVA followed by appropriate multiple-comparison tests was used. Comparisons between two groups were performed using unpaired two-tailed Student’s t-tests with Welch’s correction. Western blot signals were normalized to total protein (Direct Blue 71 staining), and proteins from mitochondrial fractions were normalized to Porin. ATP measurements were normalized to the number of fly heads and analyzed separately for males and females. Statistical tests used for individual experiments are indicated in the corresponding figure legends. A p value < 0.05 was considered statistically significant.

## Results

### SAT1 expression rescues α-Syn-induced toxicity

To assess how SAT1 modulates α-Syn-driven neurodegeneration, SAT1 and α-Syn were expressed pan-neuronally in *Drosophila* using the elav-Gal4 driver. Immunoblot analysis revealed that co-expression of SAT1 significantly reduced α-Syn protein abundance compared with α-Syn expression alone (Fig. 1b), suggesting that SAT1 influences α-Syn levels *in vivo*. To determine whether this reduction reflects altered α-Syn transcription, we measured α-Syn mRNA levels by qRT-PCR using primers specific for the human SNCA transgene. SAT1 expression did not significantly affect α-Syn transcript abundance (supplementary Fig. 1), indicating that the reduction in α-Syn protein levels is unlikely to result from decreased transgene expression. These findings suggest that SAT1 regulates α-Syn abundance through post-transcriptional mechanisms.

Following the observation that SAT1 reduces α-Syn levels, we investigated its neuroprotective potential through longevity assays in both male and female flies. Pan-neuronal expression of α-Syn drastically reduced the lifespan of both sexes (Fig. 1c, d), consistent with our previous findings using the same α-Syn *Drosophila* model(Ranxhi et al. 2025a, b). While SAT1 overexpression alone caused a marginal reduction in baseline lifespan, its co-expression with α-Syn significantly extended survival in both sexes. We observed a pronounced sexual dimorphism in the efficacy of this rescue, with female flies exhibiting a much greater lifespan extension than males (Fig. 1c, d). Female flies also consistently displayed longer lifespans in both the present study and our previous work using the same α-Syn model, suggesting an enhanced capacity to withstand proteotoxic stress. (Fig. 1c, d). Because α-Syn aggregation is a key pathological feature of PD, we next examined whether SAT1 expression impacts the subcellular distribution of α-Syn in the female fly brain (Fig. 1e, f). In flies expressing α-Syn alone, α-Syn immunoreactivity in the optic lobe was largely confined to neuronal cell bodies, with pronounced perinuclear enrichment adjacent to DAPI-labeled nuclei. In contrast, co-expression of SAT1 resulted in a broader, more diffuse α-Syn signal and a reduction in perinuclear accumulations. These data indicate that SAT1 expression is associated with altered intracellular localization of α-Syn. Consistent with these effects, neuronal SAT1 expression was associated with lower α-Syn abundance and reduced α-Syn-associated neurotoxicity *in vivo*.

### SAT1 and α-Syn drive distinct neuronal transcriptomic changes

**Fig. 2:**
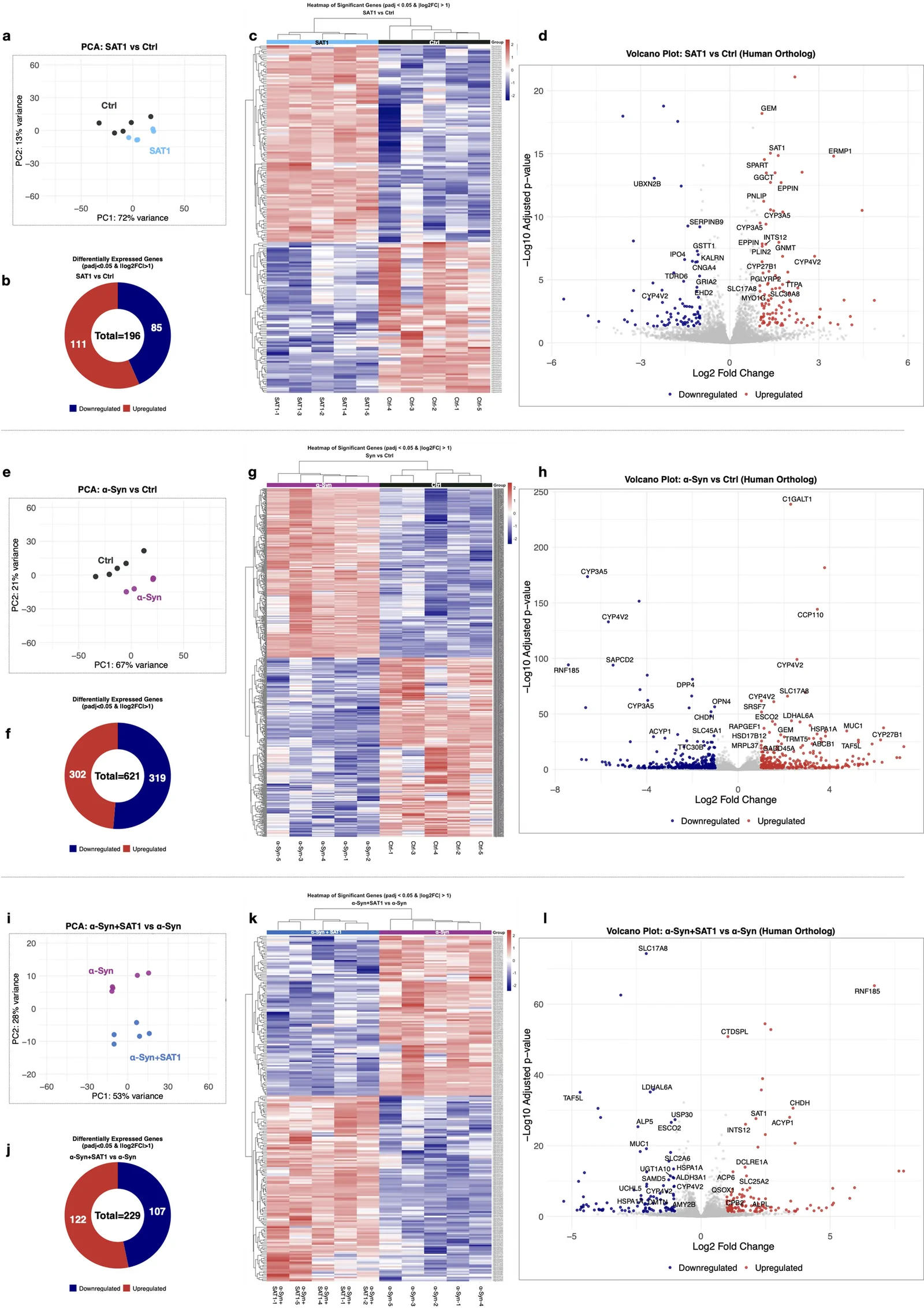
Transcriptomic analysis of SAT1 and α-Syn expression in fly heads. RNA-sequencing was performed on fly heads from four genotypes (Ctrl, SAT1, α-Syn, and α-Syn+SAT1), with five biological replicates per group. Each replicate consisted of 70 heads collected from equal numbers of male and female flies. Analyses are shown for SAT1 vs. control (**a-d**), α-Syn vs. control (**e-h**), and α-Syn+SAT1 vs. α-Syn (**i-l**). For each comparison, principal component analysis (PCA), gene count distributions of significantly upregulated and downregulated genes (adjusted *p* < 0.05, |log₂ fold change| > 1), heatmaps of differentially expressed genes, and volcano plots illustrate global and gene-specific transcriptional changes

To assess how SAT1 expression influences neuronal transcriptional responses in the presence or absence of α-Syn, we performed RNA sequencing on fly heads expressing: SAT1, α-Syn, α-Syn+SAT1 and control. A uniform analytical pipeline was applied to three biologically defined contrasts: SAT1 vs. control (Fig. 2a-d), α-Syn vs. control (Fig. 2e-h), and α-Syn+SAT1 vs. α-Syn (Fig. 2i-l). Principal component analysis (PCA) demonstrated clear genotype-dependent separation across all comparisons (Fig. 2a, e, i), indicating robust global transcriptional differences. Differential expression analysis identified substantial numbers of significantly up- and downregulated genes in each contrast (adjusted *p* < 0.05, |log₂ fold change| > 1) (Fig. 2b, f, j). Heatmaps of differentially expressed genes revealed coherent, contrast-specific expression patterns (Fig. 2c, g, k). To support biological interpretation and cross-species relevance, significantly regulated FlyBase gene identifiers were mapped to corresponding human orthologs for display in volcano plots, which illustrated the magnitude and directionality of transcriptional changes associated with SAT1 vs. control, α-Syn vs. control, and their combined expression vs. α-Syn (Fig. 2d, h, i). Collectively, these analyses demonstrate genotype-dependent transcriptional differences and establish a basis for downstream comparative and pathway enrichment analyses.

### SAT1 alters α-Syn-associated biological pathways

**Fig. 3:**
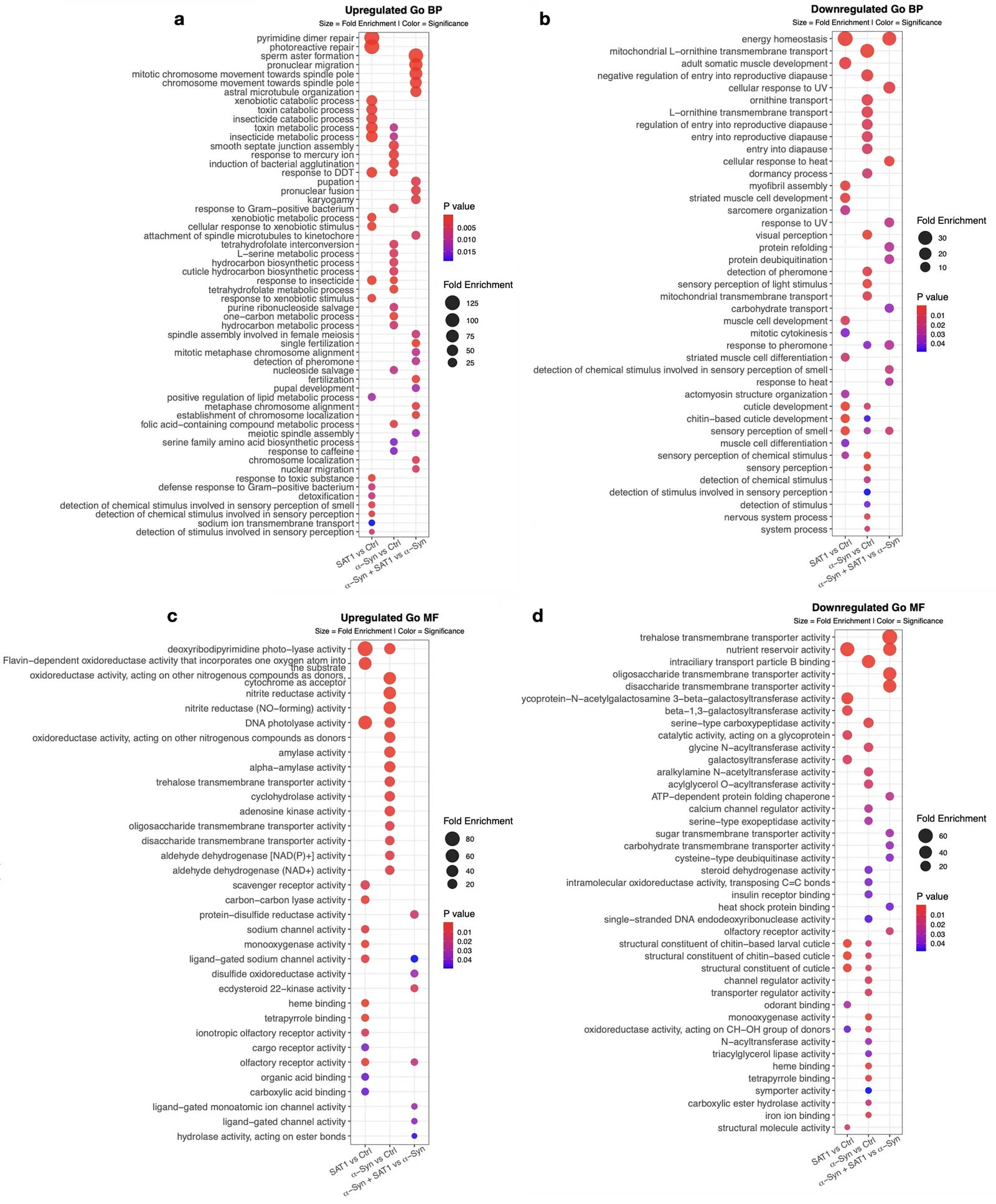
SAT1 and α-Syn differentially regulate neuronal biological pathways. Dot plots showing functional enrichment of significantly differentially expressed genes from three comparisons, SAT1 vs. control, α-Syn vs. control, and α-Syn+SAT1 vs. α-Syn. **a**, **b** GO Biological Process enrichment for upregulated (**a**) and downregulated (**b**) gene sets. **c**, **d** GO Molecular Function enrichment for upregulated (**c**) and downregulated (**d**) gene sets. Dot size indicates fold enrichment, and dot color denotes enrichment significance (p value). Only significantly enriched terms are shown

To characterize the functional categories underlying the transcriptional changes observed in Fig. 2, we performed enrichment analyses on significantly upregulated and downregulated genes from three contrasts: SAT1 vs. control, α-Syn vs. control, and α-Syn+SAT1 vs. α-Syn (Fig. 3). GO and KEGG enrichment analyses identified distinct patterns across the three contrasts.

GO Biological Process analysis (Fig. 3a, b) showed that SAT1 expression alone was associated with enrichment of pathways related to DNA repair and detoxification, accompanied by reduced representation of energy homeostasis and cytoskeletal organization. In contrast, α-Syn expression preferentially enriched xenobiotic response and detoxification pathways, as well as cellular barrier maintenance processes, while suppressing pathways associated with mitochondrial ornithine transport and dormancy. Comparison of α-Syn + SAT1 versus α-Syn revealed selective enrichment of microtubule-based transport and cytoskeletal remodeling pathways, together with reduced representation of cellular stress response and energy homeostasis pathways.

GO Molecular Function analysis (Fig. 3c, d) highlighted differences in enzymatic and binding activities across conditions. SAT1 expression alone was associated with enrichment of redox- and detoxification-related enzymatic functions, along with increased representation of receptor-mediated signaling and ion transport, whereas glycoprotein glycosylation activity and structural cuticle components were reduced. In the α-Syn condition, enrichment again centered on redox-associated enzymatic activities but extended to carbohydrate metabolism and nutrient transport, accompanied by reduced representation of acetylation-related functions, proteolysis, and membrane transport. By comparison, α-Syn+SAT1 versus α-Syn showed enrichment of redox and metabolic enzyme activities and ligand-gated ion channel-mediated neuronal signaling, together with reduced representation of carbohydrate transport, metabolic support functions, and proteostasis-linked protein quality control.

KEGG pathway analysis (supplementary Fig. 2a, b) showed that SAT1 expression alone was associated with enrichment of immune-like stress response pathways, accompanied by reduced representation of protein glycosylation, cell-surface organization, and cytoskeletal architecture. In contrast, α-Syn expression preferentially enriched pathways related to one-carbon and amino acid metabolism, carbohydrate metabolism, and O-glycan biosynthesis. Addition of SAT1 in the α-Syn background was associated with selective reduction of ascorbate and aldarate metabolic pathways and components of the basal transcriptional factors.

### SAT1 modifies the expression of genes involved in chaperone function, ubiquitination, mitochondrial biology, transcription, and transport

**Fig. 4:**
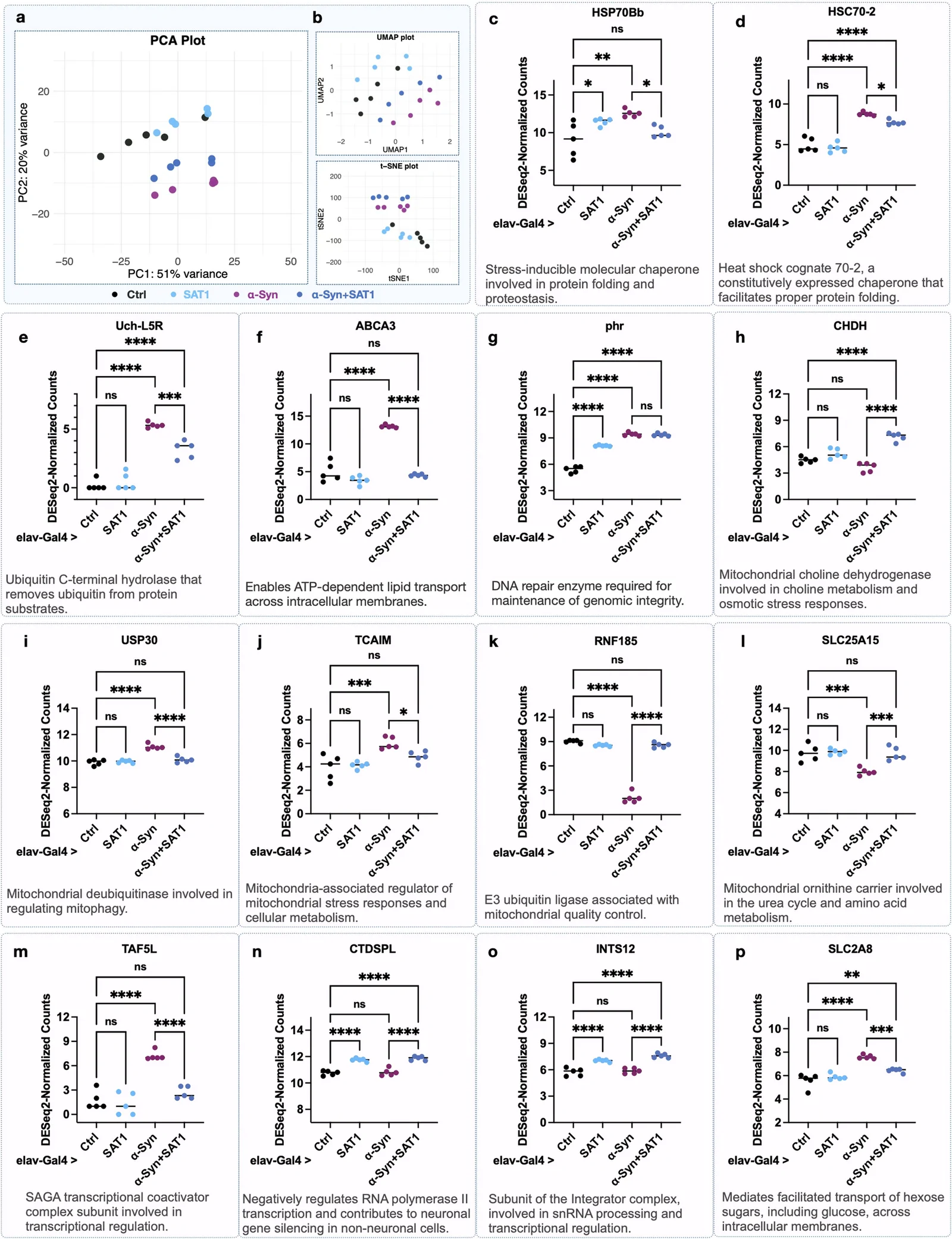
Transcriptomic profiling showed that SAT1 modulates proteostasis and stress-response pathways under α-synuclein stress. **a**, **b** Sample-level transcriptomic relationships among the four genotypes (Ctrl, SAT1, α-Syn, and α-Syn+SAT1). **a** Principal component analysis (PCA) was used as the primary dimensionality reduction approach. **b** UMAP and t-SNE were used to visualize clustering and separation of samples across groups. **c-p** Dot plots show normalized gene expression levels for selected transcripts across the four groups. Statistical significance was determined using ordinary one-way ANOVA. Significance levels: ns, not significant; * *p* < 0.05; ** *p* < 0.01; *** *p* < 0.001; **** *p* < 0.0001

To further compare global transcriptional differences across the four genotypes (control, SAT1, α-Syn, and α-Syn+SAT1), we performed principal component analysis (PCA). PCA revealed clear separation of samples along the two major axes, with PC1 accounting for 51% of the variance and PC2 accounting for 20% (Fig. 4a). Control and SAT1-alone samples clustered closely together, indicating minimal baseline transcriptional perturbation associated with SAT1 overexpression in the absence of α-Syn. In contrast, α-Syn expression produced a pronounced shift in the transcriptome along a distinct direction. Notably, co-expression of SAT1 with α-Syn formed a separate cluster from α-Syn alone, indicating that SAT1 modifies the transcriptional response to α-Syn expression rather than simply restoring it toward baseline. Nonlinear dimensionality reduction using UMAP and t-SNE yielded similar separation patterns and confirmed high consistency among biological replicates (Fig. 4b).

We next focused on genes significantly altered by α-Syn expression and examined how their mRNA levels changed across the four genotypes (control, SAT1, α-Syn, and α-Syn+SAT1) to assess the impact of SAT1 co-expression (Fig. 4c-p). SAT1 overexpression alone had minimal effects on most genes examined, including the stress-inducible chaperone *HSP70Bb* (Fig. 4c) (Singh et al. 2025), the constitutive chaperone *HSC70-2* (Fig. 4d) (Pemberton et al. 2011), and the ubiquitin hydrolase *Uch-L5R* (Fig. 4e) (Zhang et al. 2024). In contrast, α-Syn expression was associated with robust upregulation of these transcripts. Co-expression of SAT1 attenuated this induction, shifting mRNA levels toward those observed in control samples. A similar pattern was observed for *ABCA3*, an ATP-dependent lipid transporter (Piehler et al. 2012) elevated by α-Syn and reduced upon SAT1 co-expression (Fig. 4f). In contrast, expression of *phr*, a DNA photolyase involved in genomic maintenance (Ozturk 2017), was increased by both SAT1 and α-Syn, with additive effects observed upon co-expression (Fig. 4g).

Several genes associated with mitochondrial quality control also showed pronounced regulation in response to α-Syn expression, with their mRNA levels modified by SAT1 co-expression. These included *CHDH*, a mitochondrial enzyme (Li et al. 2023) involved in osmoprotection and metabolism (Fig. 4h); *USP30* (Siwach et al. 2025), a deubiquitinase that negatively regulates mitophagy (Fig. 4i); *TCAIM*, a regulator of mitochondrial stress responses (Fig. 4j) (Jiahui et al. 2025); *RNF185*, an E3 ubiquitin ligase implicated in mitochondrial surveillance (Fig. 4k) (Tang et al. 2011); and *SLC25A15*, a mitochondrial ornithine carrier linked to urea cycle and amino acid metabolism (Fig. 4l) (Ersoy Tunali et al. 2014). Together, these expression changes indicate that SAT1 co-expression is associated with altered regulation of mitochondrial-related pathways engaged by α-Syn expression.

In addition, SAT1 co-expression affected several transcriptional regulators, including *TAF5L* (Fig. 4m), a component of the SAGA complex (Helmlinger and Tora 2017); *CTDSPL* (Fig. 4n), a CTD phosphatase that negatively regulates RNA polymerase II transcriptional activity (Krasnov et al. 2019); and *INTS12* (Fig. 4o), a subunit of the Integrator complex involved in transcriptional pausing and RNA processing (Chen et al. 2013). Finally, expression of *SLC2A8* (Fig. 4p), a facilitative glucose and fructose transporter (Schmidt et al. 2008) strongly induced by α-Syn, was reduced upon SAT1 co-expression.

To further assess whether SAT1 counter-regulates α-Syn-associated transcriptional changes, we performed a reversal analysis integrating results from the α-Syn vs. control and α-Syn+SAT1 vs. α-Syn comparisons (supplementary Fig. 3). For each gene, log₂ fold changes from the α-Syn vs. control comparison were plotted on the x-axis and log₂ fold changes from the α-Syn+SAT1 vs. α-Syn comparison were plotted on the y-axis. Genes downregulated by α-Syn and upregulated upon SAT1 co-expression localized to the upper-left quadrant, whereas genes upregulated by α-Syn and suppressed by SAT1 localized to the lower-right quadrant. These reciprocal patterns indicate that SAT1 selectively counter-regulates specific α-Syn-associated transcriptional changes rather than globally restoring gene expression toward control levels.

### SAT1 enhances mitochondrial LC3 association and reduces mitochondrial α-Syn accumulation

Our RNA-seq analyses indicated that genes associated with mitochondrial stress responses and quality-control pathways are prominently regulated in the context of α-Syn expression and are modified by SAT1 co-expression, suggesting mitochondria as a potential point of convergence between α-Syn toxicity and SAT1-associated protection. To directly examine mitochondrial alterations *in vivo*, we analyzed mitochondrial organization and autophagy-related features in female fly brains expressing α-Syn alone or α-Syn together with SAT1.

**Fig. 5:**
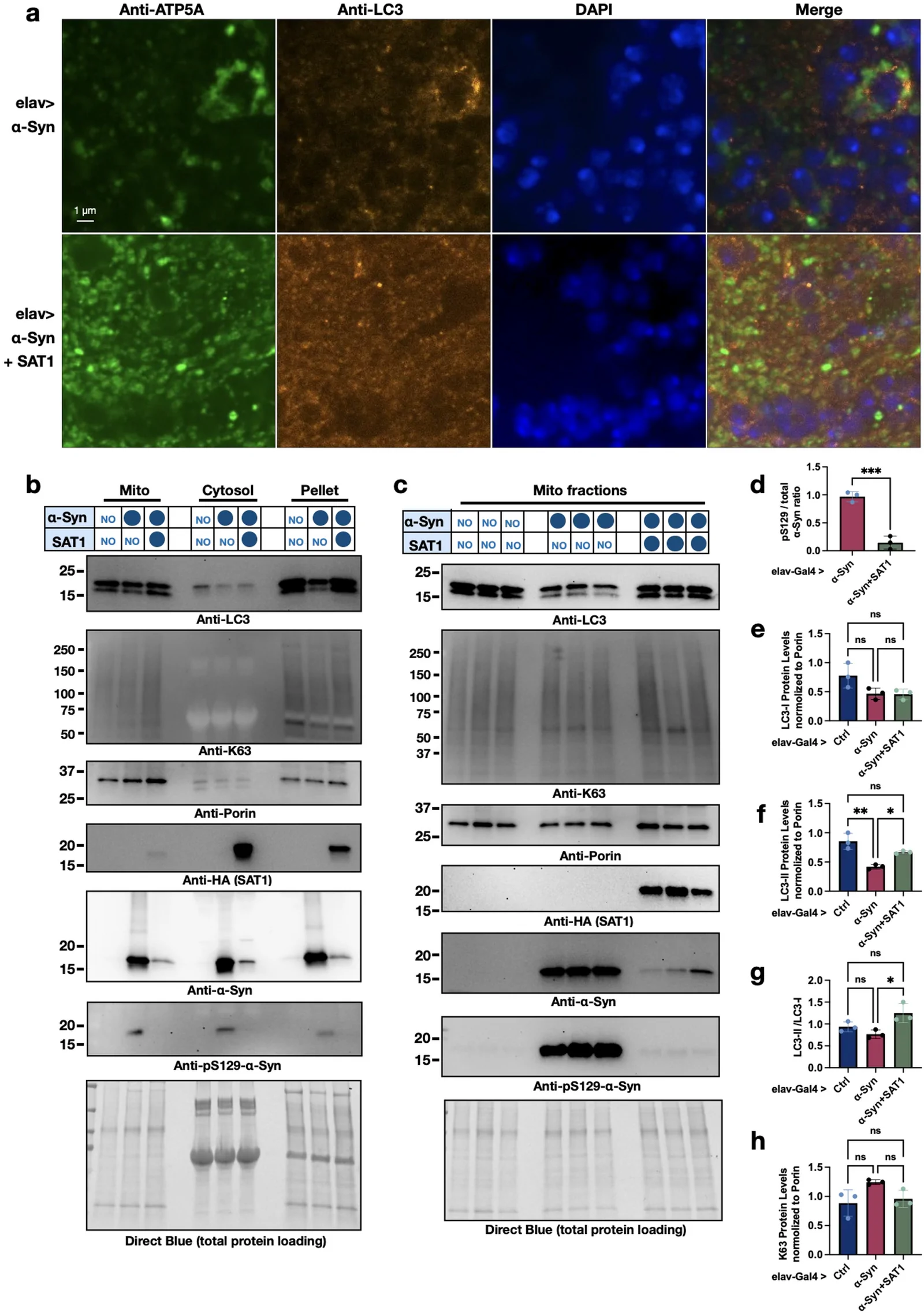
SAT1 co-expression enhances mitochondrial ATP5A abundance and LC3 activation in α-Syn expressing brains. **a** Confocal images of female fly brains immunostained for ATP5A (green), LC3 (orange), and DAPI (blue). Scale bar, 1 μm. (b-c) Western blot analysis of subcellular fractions, including **b** mitochondrial, cytosolic, and pellet fractions and **c** isolated mitochondrial fractions, probed for LC3, K63-linked ubiquitin, Porin, HA-SAT1, α-Syn, pS129-α-Syn, and total protein (Direct Blue). Each biological replicate consisted of 50 fly heads collected from equal numbers of male and female flies (25 males and 25 females). **d-h** Quantification of the indicated protein levels from the mitochondrial fractions shown in (**c**). Statistical comparisons were performed using ordinary one-way ANOVA. Significance levels: ns, not significant; * *p* < 0.05; ** *p* < 0.01; *** *p* < 0.001

Using the mitochondrial marker ATP5A and the autophagy marker LC3, α-Syn-expressing neurons exhibited reduced ATP5A signal and relatively sparse LC3 puncta (Fig. 5a). In contrast, co-expression of SAT1 was associated with increased ATP5A intensity and more abundant LC3 puncta throughout the neuropil. Merged images showed increased spatial overlap between ATP5A and LC3 in α-Syn+SAT1 brains, consistent with enhanced association of autophagy-related markers with mitochondrial compartments.

We next isolated mitochondrial fractions to further examine the subcellular distribution of SAT1, α-Syn, and autophagy-related markers (Fig. 5b). Fractionation separated cytosolic, pellet, and mitochondrial-enriched fractions. SAT1 protein was detected predominantly in the cytosolic and pellet fractions, and was not enriched in the mitochondrial fraction. In contrast, α-Syn was detected across all three fractions, and SAT1 co-expression was associated with reduced α-Syn abundance in each fraction, similar to our observations of SAT1-depedent effects on total α-Syn protein levels. Focusing on the mitochondrial fraction, α-Syn levels were lower in SAT1 + α-Syn samples compared with α-Syn alone (Fig. 5c), indicating reduced mitochondrial-associated α-Syn when SAT1 is present.

To determine whether SAT1 also affects pathological α-Syn species, we examined α-Syn phosphorylated at Ser129 (pS129) (Fig. 5b, c). Similar to total α-Syn, mitochondrial pS129 α-Syn levels were markedly reduced in the presence of SAT1 (Fig. 5c). Quantitative analysis revealed that the decrease in pS129 α-Syn exceeded that observed for total α-Syn, resulting in a lower pS129/total α-Syn ratio (Fig. 5d). These findings suggest that SAT1 co-expression preferentially limits the accumulation of phosphorylated α-Syn species associated with mitochondria.

Analysis of autophagy markers in mitochondrial fractions revealed that α-Syn expression was associated with reduced total LC3 abundance, whereas SAT1 co-expression increased LC3 levels (Fig. 5c). After normalization to porin, which reflects mitochondrial content, LC3-I levels were not appreciably altered across groups (Fig. 5e). In contrast, LC3-II levels were reduced in α-Syn-expressing samples and showed partial recovery upon SAT1 co-expression (Fig. 5f). Consistent with this, the LC3-II/LC3-I ratio, which declined with α-Syn expression, was increased by SAT1 co-expression (Fig. 5g). Levels of K63-linked ubiquitin, a marker associated with selective autophagy and mitochondrial tagging, did not differ significantly across conditions (Fig. 5h).

These data indicate that SAT1 co-expression is associated with reduced accumulation of mitochondrial α-Syn, particularly phosphorylated α-Syn species, and increased levels of membrane-associated LC3 (LC3-II), consistent with altered engagement of mitochondrial-associated quality-control processes during α-Syn-associated stress.

### SAT1 preserves cellular energy homeostasis in α-Syn-expressing flies

**Fig. 6:**
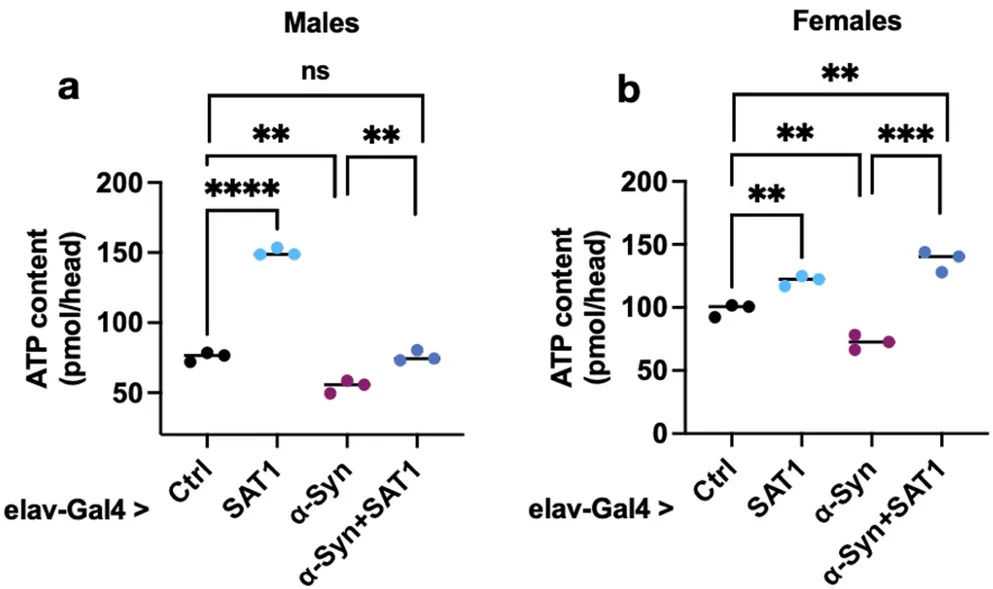
SAT1 preserves cellular ATP levels in α-Syn-expressing flies. **a**, **b** Steady-state ATP content measured by a luciferase-based bioluminescence assay in **a** male and **b** female fly heads from control, SAT1, α-Syn, and α-Syn + SAT1 genotypes. ATP levels are presented as pmol ATP per fly head. Each biological replicate consisted of 10 fly heads from the indicated sex, with three independent biological replicates per group. Statistical significance was determined using ordinary one-way ANOVA. Significance levels: ns, not significant; * *p* < 0.05; ** *p* < 0.01; *** *p* < 0.001; **** *p* < 0.0001

To determine whether the transcriptional changes and increased mitochondrial marker signal were accompanied by functional alterations in cellular energy homeostasis, we quantified steady-state ATP levels in adult fly heads. Neuronal overexpression of SAT1 alone significantly increased ATP content in both males and females (Fig. 6a, b). In contrast, α-Syn expression significantly reduced ATP levels in both sexes. Co-expression of SAT1 significantly attenuated the α-Syn-induced ATP deficit (*p* < 0.01 in males; *p* < 0.001 in females). In males, ATP levels were restored to values comparable to controls (Fig. 6a), whereas in females, ATP levels exceeded those observed in control flies (Fig. 6b). Interestingly, the more pronounced ATP restoration observed in female flies mirrors the greater lifespan extension previously observed in α-Syn-expressing females. These findings provide functional support for the transcriptomic, imaging, and biochemical evidence implicating mitochondrial pathways and suggest that SAT1 helps maintain cellular energy homeostasis during α-Syn-induced proteotoxic stress.

## Discussion

Polyamine metabolism has emerged as an important determinant of cellular stress responses, with spermidine/spermine N¹-acetyltransferase 1 (SAT1) occupying a central position in polyamine interconversion and turnover (Pegg 2008). In this study, we identify SAT1 as a robust modifier of α-Syn-related neurotoxicity and outline molecular and cellular features associated with SAT1-mediated protection in vivo. Neuronal SAT1 overexpression mitigated organismal consequences of α-Syn expression, including improved survival, and was associated with reduced α-Syn protein levels and altered α-Syn distribution within the nervous system, consistent with observations from mammalian model systems (Lewandowski et al. 2010; Zahedi et al. 2020). Similar findings were observed in our previous study using the same *Drosophila* α-Syn model, where neuronal SAT1 knockdown increased α-Syn levels and exacerbated α-Syn-associated phenotypes, whereas SAT1 overexpression produced the opposite effects (Ranxhi et al. 2025b). These complementary gain- and loss-of-function findings support a specific role for SAT1 in modulating α-Syn toxicity.

Neuronal SAT1 overexpression alone resulted in a modest reduction in baseline lifespan, particularly in males, suggesting that maintaining polyamine homeostasis within a narrow physiological range is important for normal neuronal function. Chronic activation of polyamine catabolism may impose a mild physiological burden under basal conditions by perturbing intracellular polyamine balance. In contrast, the marked survival benefit observed in α-Syn-expressing flies indicates that the effects of SAT1 are highly context dependent. In the setting of α-Syn-induced proteotoxic stress, the reduction in α-Syn accumulation and preservation of mitochondrial homeostasis associated with SAT1 expression appear to outweigh the modest physiological costs of sustained SAT1 overexpression.

The magnitude of this protection was greater in females than in males. Female flies consistently exhibited longer lifespans in this and our previous studies using the same α-Syn model (Ranxhi et al. 2025b), suggesting an enhanced capacity to withstand proteotoxic stress. Although the mechanisms underlying this sexual dimorphism remain unclear, males and females likely differ in their metabolic responses and stress-adaptive pathways. These observations are consistent with the well-recognized sex differences observed in Parkinson’s disease (Willis et al. 2022) and suggest that the effects of polyamine metabolism on neuronal resilience may be influenced by sex. However, the present study was not designed to define the mechanisms responsible for these sex-dependent effects. Future studies examining the interplay between sex, polyamine regulation, and neurodegenerative pathways may provide further insight into the basis of SAT1-mediated neuroprotection.

Transcriptomic analysis suggests that SAT1 co-expression modulates neuronal responses to α-Syn stress, rather than simply restoring gene expression to baseline. SAT1 reduced activation of stress-responsive and energy-consuming pathways, while enhancing expression of genes involved in cytoskeletal organization and neuronal signaling. This pattern is consistent with a compensatory shift in which persistent stress is suppressed and cellular resources are redirected to support structural maintenance and synaptic function. This outcome aligns with the known roles of polyamines in translation, nucleic acid binding, and metabolic regulation (Pegg 2006).

Multiple lines of evidence point to mitochondria as a relevant downstream intersection between α-Syn toxicity and SAT1-associated protection. A subset of α-Syn-responsive genes modified by SAT1 encode proteins involved in mitochondrial metabolism and quality control, including CHDH, USP30, TCAIM, RNF185, and the mitochondrial ornithine carrier SLC25A15. While transcriptional changes alone do not establish causality, the coordinated regulation of these genes suggests that SAT1 influences neuronal responses to mitochondrial stress induced by α-Syn. Supporting this interpretation, imaging and biochemical analyses revealed reduced mitochondrial marker signal and limited LC3 association in α-Syn-expressing brains, whereas SAT1 co-expression was associated with increased ATP5A signal, increased LC3 puncta, and greater spatial overlap between mitochondrial and autophagy markers. These observations support altered engagement of mitochondrial quality-control pathways under conditions in which SAT1 attenuates α-Syn toxicity.

ATP measurements further supported these findings. SAT1 expression increased steady-state ATP levels under basal conditions and attenuated the ATP deficit induced by α-Syn expression. This effect was particularly evident in females, paralleling the greater lifespan extension observed in female α-Syn flies. Although ATP content alone does not distinguish between altered ATP production and consumption, these data provide functional evidence that SAT1 mitigates energetic deficits associated with α-Syn-induced stress. Additional studies employing measurements of mitochondrial respiration and membrane potential will be necessary to define the precise bioenergetic mechanisms underlying these effects.

Functional enrichment analyses further suggest that α-Syn expression disrupts metabolic pathways linking mitochondrial amino acid handling to cytosolic polyamine homeostasis, including reduced expression of genes involved in mitochondrial ornithine transport. Mitochondrial ornithine transport coordinates amino acid metabolism, nitrogen flux, and polyamine biosynthesis (Fiermonte et al. 2003; Monne et al. 2015); its suppression is therefore expected to uncouple mitochondrial metabolism from cellular control of polyamine balance, increasing vulnerability to metabolic and proteotoxic stress. Notably, SAT1 co-expression was associated with restoration of SLC25A15 expression in the α-Syn background, indicating that SAT1 can influence this component of mitochondrial amino acid transport under stress conditions.

Moreover, SAT1 can modulate polyamine pools directly through acetylation of spermidine and spermine (Pegg 2009), limiting accumulation of higher-order polyamines and facilitating their export or turnover (Casero and Marton 2007; Pegg 2008). These observations suggest that SAT1 may act at multiple levels to stabilize polyamine homeostasis during α-Syn-induced stress, both by influencing mitochondrial amino acid transport and by adjusting intracellular polyamine composition. Although our data do not establish whether regulation of SLC25A15 is direct or secondary to broader metabolic remodeling, the coordinated transcriptional, biochemical, and functional changes observed here support an association between SAT1 activity and improved integration of mitochondrial metabolism with polyamine homeostasis during α-Syn expression.

Although degeneration of dopaminergic neurons is a pathological hallmark of Parkinson’s disease, α-Syn pathology is not restricted to this neuronal population and may emerge in extranigral regions before substantia nigra degeneration (Braak et al. 2003; Burke et al. 2008). Increasing evidence indicates that selective neuronal vulnerability in PD is influenced not only by intrinsic properties of dopaminergic neurons but also by the broader neuronal network and neighboring cell populations that support their survival (Alegre-Abarrategui et al. 2019; Gonzalez-Rodriguez et al. 2021). Accordingly, the pan-neuronal approach used in the present study provides insight into neuronal responses that may influence selective vulnerability. Nevertheless, the current study does not address cell type-specific effects of SAT1. Future studies employing dopaminergic neuron-specific models will be required to determine whether SAT1-mediated protection extends to these particularly susceptible neurons and to define how polyamine metabolism interacts with cell type-specific mechanisms governing neuronal survival.

In conclusion, these findings place polyamine interconversion at the intersection of mitochondrial amino acid transport, cellular stress regulation, and neuronal resilience under α-Syn-induced stress. While our data define robust associations among SAT1 expression, reduced α-Syn-associated toxicity, transcriptional modulation, and mitochondrial and autophagy-linked signatures, additional studies will be required to establish causal relationships. Nonetheless, this work identifies SAT1 as a functionally relevant regulator of neuronal stress adaptation and highlights polyamine metabolism as a biologically grounded axis influencing vulnerability in synucleinopathy-related neurodegeneration.

## Supplementary Information

Below is the link to the electronic supplementary material.

Supplementary Material 1

## Data Availability

Fly lines and source data are available upon request. The authors affirm that all data necessary for confirming the conclusions of the article are present within the article and figures. To request data from this study, please contact W-L.T at [wtsou@wayne.edu](mailto: wtsou@wayne.edu) . Due to the temporary unavailability of GEO submission services at NCBI, RNA-seq raw data and processed differential expression matrices are available upon reasonable request and will be deposited in a public repository upon restoration of submission access. All R scripts used for data processing and visualization were generated in RStudio (Boston, MA, USA) and are available upon request. Requests for data and code should be directed to W-L.T.
