# Supplementary material for: Polyamine metabolic enzyme SAT1 remodels the neuronal transcriptome and rescues α-synuclein toxicity in Drosophila
Source: Amino Acids. 2026 Aug 30;58:48. doi: 10.1007/s00726-026-03544-y (PMC13575090; doi:10.1007/s00726-026-03544-y)

Polyamine metabolic enzyme SAT1 remodels the neuronal transcriptome and rescues α-synuclein toxicity in *Drosophila*

Zoya R. Bangash[1], Hiroyoshi Matsui[1], Bedri Ranxhi[1], Sokol V. Todi[1,2], Peter A. LeWitt*[1,2], Wei-Ling Tsou*[1]

1- Department of Pharmacology, Wayne State University School of Medicine, Detroit, MI, U.S.A.
2- Department of Neurology, Wayne State University School of Medicine, Detroit, MI, U.S.A.

Supplementary material

a

SNCA.J nucleotide sequence

ATCAAC**ATG**GACGTATTCATGAAGGGTTTGTCCAAAGCCAAAGAAGGAGTCGTGGCCGCTGCC
GaaaAGACGAAGCAGGGTGTGGCGGAGGCGGCTGGCAAAACCAAGGAGGGAGTTCTCTACG
TGGGCAGTAAGACTAAGGAGGGTGTGGTGCACGGCGTCGCCACCGTGGCAGAAAAGACCAA
GGAGCAGGTGACAAATGTGGGAGGCGCCGTTGTTACCGGCGTGACTGCCGTCGCCCAAAAG
ACAGTAGAAGGCGCTGGTTCGATCGCGGCAGCCACGGGCTTTGTCAAGAAGGATCAGCTGGG
CAAAAACGAGGAGGGAGCGCCCCAAGAGGGAATTCTTGAGGACATGCCCGTCGATCCAGACA
ACGAGGCTtACGAGATGCCTAGCGAAGAGGGATATCAGGATTACGAACCGGAGGCATAA

b

Amino acid alignment

```
Human α-Syn                1 MDVFMKGLSKAKEGVVAAAEKTKQGVAEAAGKTKEGVLYVGSKTKEGVVH     50
                             ||||||||||||||||||||||||||||||||||||||||||||||||||
SNCA.J (codon-optimized)   1 MDVFMKGLSKAKEGVVAAAEKTKQGVAEAAGKTKEGVLYVGSKTKEGVVH     50

Human α-Syn               51 GVATVAEKTKEQVTNVGGAVVTGVTAVAQKTVEGAGSIAAATGFVKKDQL    100
                             ||||||||||||||||||||||||||||||||||||||||||||||||||
SNCA.J (codon-optimized)  51 GVATVAEKTKEQVTNVGGAVVTGVTAVAQKTVEGAGSIAAATGFVKKDQL    100

Human α-Syn              101 GKNEEGAPQEGILEDMPVDPDNEAYEMPSEEGYQDYEPEA    140
                             ||||||||||||||||||||||||||||||||||||||||
SNCA.J (codon-optimized) 101 GKNEEGAPQEGILEDMPVDPDNEAYEMPSEEGYQDYEPEA    140
```

c

Primers:

SNCA.J-1F CAAAGCCAAAGAAGGAGTCGTG
SNCA.J-1R CACATTTGTCACCTGCTCCTTG

rp49-F AGATCGTGAAGAAGCGCACCAAG
rp49-R CACCAGGAACTTCTTGAATCCGG

d

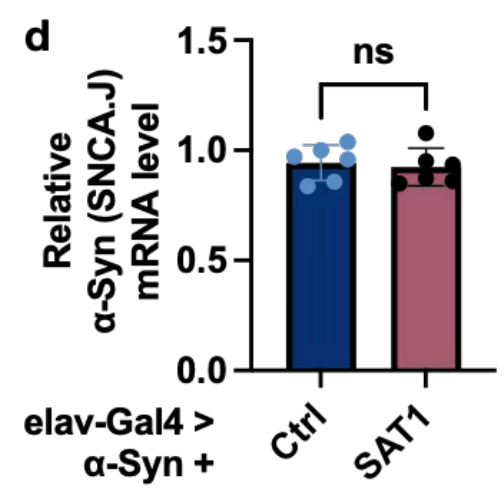

Supplementary fig. 1. Validation of the UAS-SNCA.J (α-Syn) transgene and analysis of α-Syn transcript levels.

(a) Nucleotide sequence of the insect codon-optimized human SNCA transgene (UAS-SNCA.J; referred to as α-Syn throughout the manuscript) used in this study. (b) Amino acid sequence alignment between human α-Syn and the translated UAS-SNCA.J transgene product. Vertical bars indicate identical amino acid residues. Despite nucleotide codon optimization, the transgene encodes a protein sequence identical to human α-Syn (140 amino acids, 100% identity). (c) Sequences of qRT-PCR primers designed to specifically amplify the human SNCA.J transgene. (d) Quantitative RT-PCR analysis of α-Syn (SNCA.J) transcript levels in fly heads expressing α-Syn alone or α-Syn together with SAT1. SAT1 expression did not significantly alter α-Syn transcript levels, indicating that the reduction in α-Syn protein observed in SAT1-expressing flies is not attributable to decreased transgene transcription. Data are presented as mean ± SEM. Statistical significance was determined using an unpaired two-tailed Student's t-test.

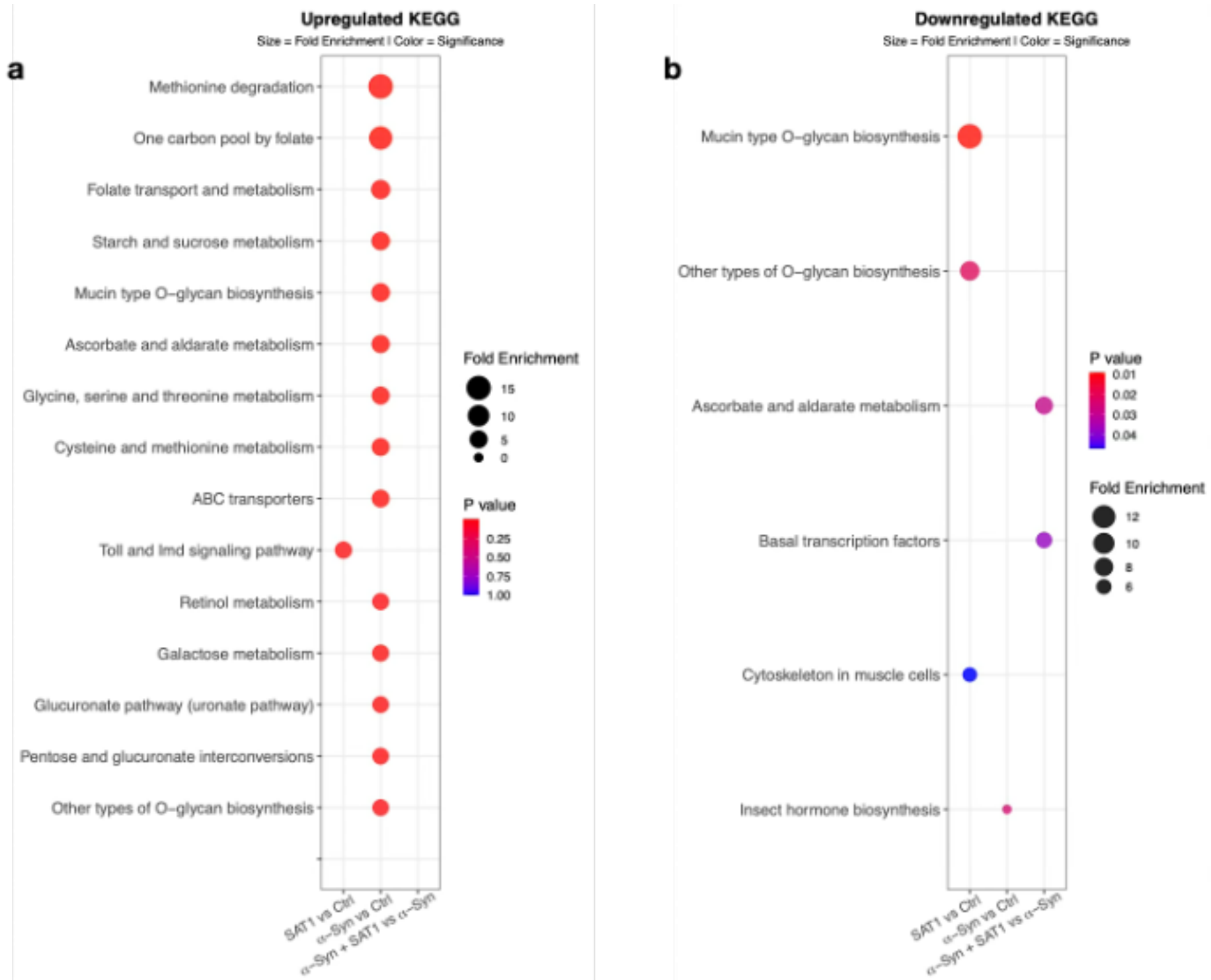

Supplementary fig. 2.

SAT1 and α-Syn differentially regulate neuronal biological pathways.

Dot plots showing functional enrichment of significantly differentially expressed genes from three comparisons, SAT1 vs control, α-Syn vs control, and α-Syn+SAT1 vs α-Syn. KEGG pathway enrichment for upregulated (a) and downregulated (b) gene sets. Dot size indicates fold enrichment, and dot color denotes enrichment significance (p value). Only significantly enriched terms are shown.

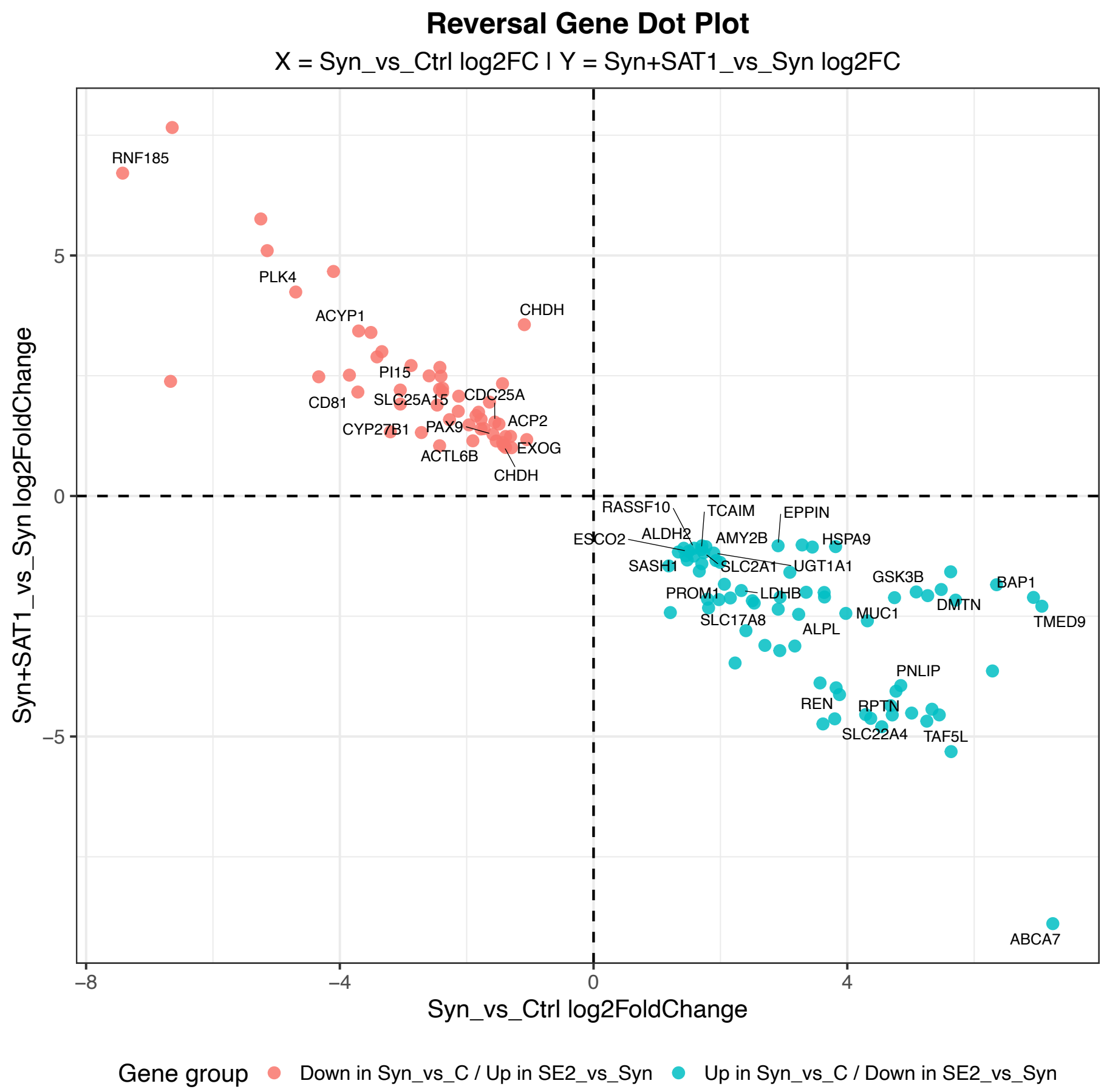

Supplementary fig. 3.

Reversal of α-Syn-associated gene expression by SAT1 co-expression.

To identify genes whose α-Syn-associated expression changes were reversed by SAT1 co-expression, DESeq2 results from the α-Syn versus control and α-Syn+SAT1 versus α-Syn comparisons were integrated. Each gene is plotted by its $\log_2$ fold change in α-Syn versus control (x-axis) and α-Syn+SAT1 versus α-Syn (y-axis). Genes were required to be significantly differentially expressed in both contrasts (adjusted p value < 0.05; $|\log_2$ fold change$| > 1$).

Genes downregulated by α-Syn but upregulated upon SAT1 co-expression are shown as salmon-colored dots and cluster in the upper-left quadrant. Genes upregulated by α-Syn but suppressed by SAT1 are shown as teal-colored dots and cluster in the lower-right quadrant. Dashed lines indicate zero $\log_2$ fold change in each comparison. Gene-level results were merged by FlyBase gene identifier and annotated with corresponding human ortholog symbols for visualization.

Supplementary fig. 4.

Uncropped Blots

Figure 1b

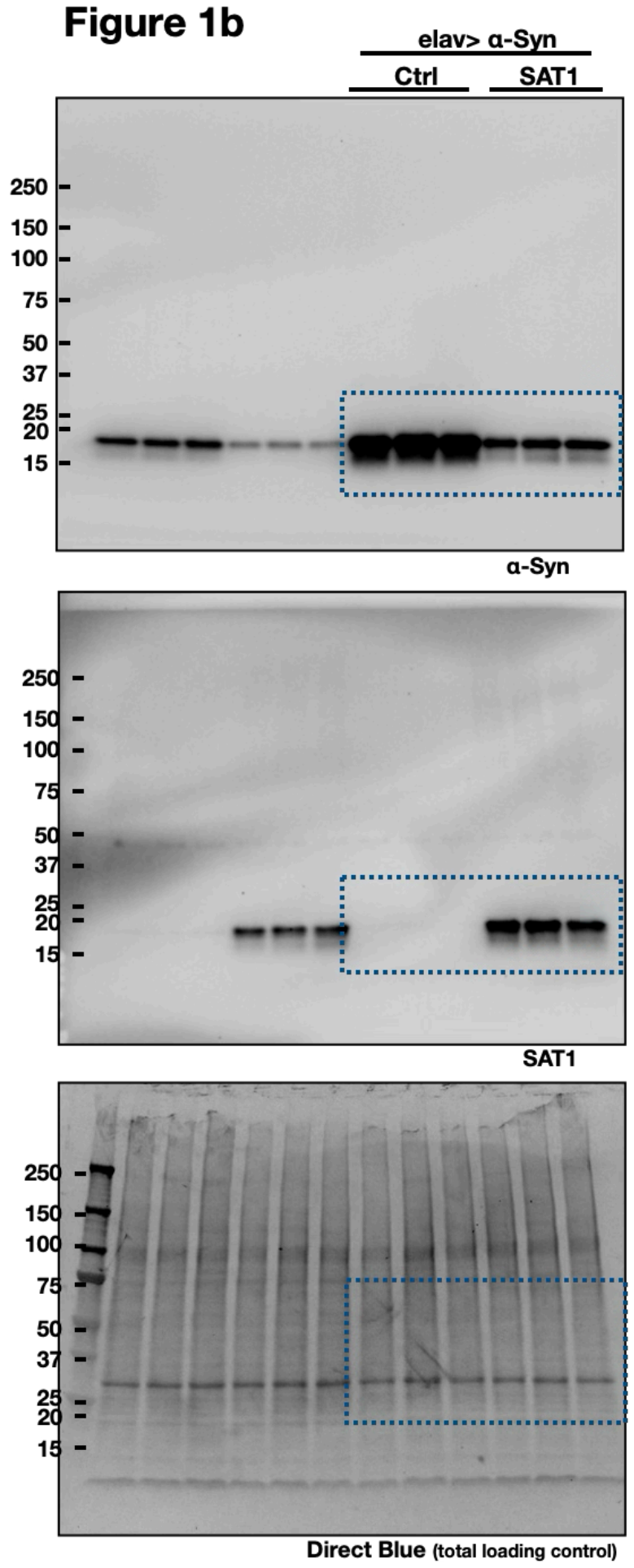

Figure 5b

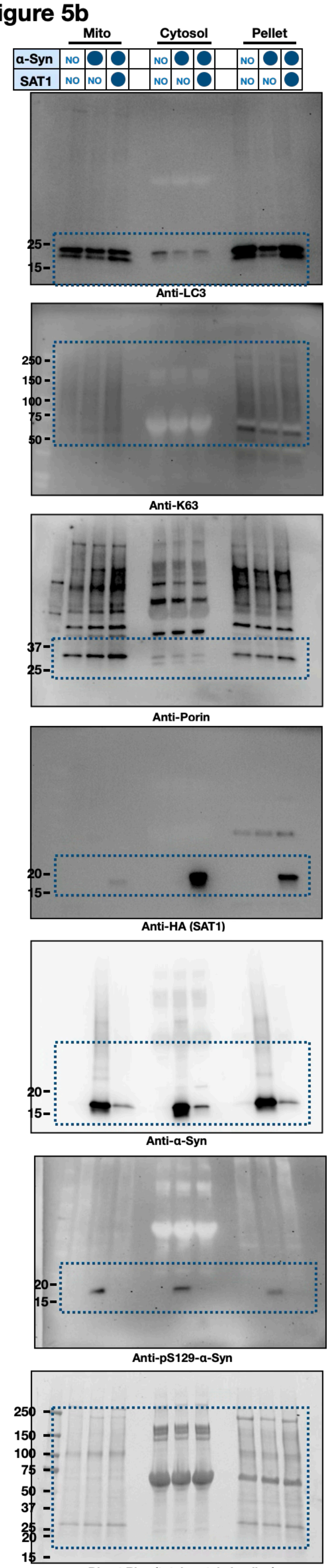

Figure 5c

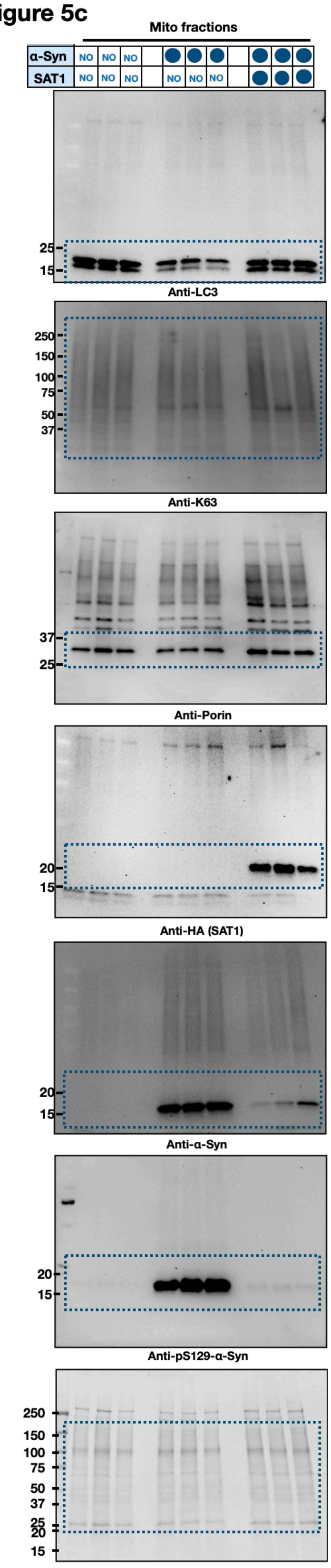

Supplement: Supplementary file 1 [file 726_2026_3544_MOESM1_ESM.pdf]
